# Detecting formal and informal interests in forest governance

**DOI:** 10.1016/j.mex.2022.101842

**Published:** 2022-09-05

**Authors:** Jiacheng Zhao, Max Krott, Jinlong Liu, Lukas Giessen, Jiayun Dong

**Affiliations:** a Institute of Forest and Nature Conservation Policy, University of Goettingen, Germany; b School of Agriculture and Rural Development, Renmin University of China, China; c Chair of Tropical and International Forestry, TU Dresden, Germany; d School of Economics and Management, Nanjing Forestry University, China

**Keywords:** Qualitative research, Forest policy, Forest governance, Environmental politics, Nature conservation policy

## Abstract

To reveal the interests of actors in forest governance, this paper proposes a power-based interest identification (PII) approach. Based on the assumption of intentional action, the benefits that actors derive from policy impacts are the result of interest-driven actions. This paper further proposes a theoretical definition of interests that includes formal goals at the social and ecological levels, as well as informal political, economic, and strategic interests. Researchers need to identify powerful actors by identifying power mechanisms and resources, and can then observe actors' formal goals through interviews and documents. For informal interests, the actor observes the informal gains of powerful actors in policy impacts, which are then coded according to political, economic, and strategic interests. Combining these steps, actors can infer the formal and informal interests of powerful actors.

• Researchers can verify actors’ formal objectives by interview and documents.
• Among policy impacts, researchers can observe influences at the social and ecological levels, as well as changes in actors' control, economic gains, and dissemination of ideas.
• Researchers can infer informal interests of powerful actors from observation of policy impacts.

## Graphical abstract

**Figure:**
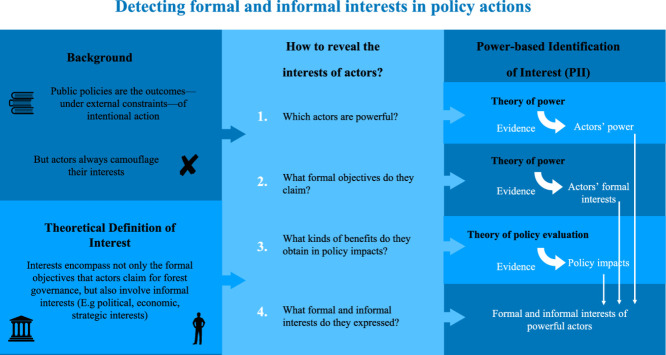

Specifications table

**Table:** 

Subject area:	Environmental Science
More specific subject area:	Forest Policy and Governance
Name of your method:	Power-based Identification of Interest
Name and reference of original method:	Not applicable
Resource availability:	Not applicable

## Method details

### Background

"Not directly with the eye, but the effects are visible". (Roentgen, 1893)

Both the formulation and implementation of forest policy are profoundly influenced by the conflicting interests of actors [1,2]. However, actors always camouflage their informal interests [3,4]. Given that the researcher cannot verify the authenticity of the information, directly inquiring about the interests of the actors is not a feasible option [4,5]. In existing studies, the identification of interests is either based on theoretical assumptions [6,7] or dependent on the experience of the actors [2,8]. Therefore, finding a way to reveal the actors' interests is an urgent challenge.

Scharpf points out that “public policies are the outcomes—under external constraints—of intentional action [9].” Therefore, the benefits of actors in the outcome of forest policy can be considered as the result of an interest-driven game [10]. This article proposes that the unclaimed interests of actors can be revealed by observing their benefits in policy impacts. Based on empirical evidence, researchers are expected to answer the following questions. Who are the powerful actors? What formal objectives do they claim? What benefits do they gain in policy impacts? What are the formal and informal interests expressed by the powerful actors? This paper refers to the above approach as power-based interest identification (PII).

### Theoretical definition of interest

The theoretical definition of interest underpins the PII approach and provides the researcher with the tools to code in the subsequent steps. This article adopts Krott's definition of interests which “are based on action orientation, adhered to by individuals or groups, and they designate the benefits the individual or group can receive from a certain object, such as a forest [10].” Interests encompass not only the formal objectives that actors claim for forest governance [8], but also involve actors' self-interests [10]. Formal objectives are “normative expectations addressed to the occupants of given positions [9].” They are declared by individuals and organizations at both the social and ecological dimensions [11]. Social objectives address issues such as economic development, empowerment, and equity for human entities [11,12]. And ecological objectives relate to topics such as resource use and ecosystem protection [5,12].

Informal interests are linked with rational self-interested action [9]. Inspired by the research of Rahman and Giessen [8] and Sahide et al. [13], this article distinguishes the political, economic, and strategic interests of actors at the informal level. It needs to be mentioned that actors not only expand their interests in the process of policy development and implementation, but also use policy to gain benefits from external actors. For example, government officials always want to expand their influence in forest conservation projects, and some of them also hope to use the project's performance to gain opportunities for promotion.

Political interests refer to the desire of actors to maximize their influence. As power is man's ultimate goal [14], actors strive to expand their influence in policy formulation and implementation. Economic interest refers to the actors’ desire to maximize their economic income. This comprises not only the expectations of forest users for timber output [2,10], but also the desire of organizations for budgets and fundraising [15]. Strategic interest refers to the desire of actors to spread their favored ideas. Strategic interests emphasize the values or ideologies, except for formal objectives, of individuals and organizations. For example, bureaucratic organizations always develop and maintain their own ideologies [15], the World Bank has been advocating neoliberalism and good governance [8], and local communities prefer to retain indigenous knowledge [16].

### Identification of powerful actors’ interests

In line with the assumption of intentional action, it is necessary to clarify two scopes of applying the PII approach. First, the PII approach aims at observing interests of the powerful. Under the guarantee of power, the potentate can achieve its interest which is expressed in the change in the policy impact. On the contrary, the benefits of the subordinate are hardly probed in policy impact. Second, according to this assumption, the PII approach requires a stable external condition. Thus, researchers should check the stability of actors' networks and their resources in the policy process. Then, researchers may seek clues to the classification of actors, resources and interests through existing theories. For example, researchers can adopt the theory of bureaucratic politics as a starting point for understanding the development of forest policies.

#### Step 1: Observing actors’ power

This article divides the PII approach into four steps shown in Table 1. In the first step, what needs to be identified primarily is the network of actors. The researcher can use the existing knowledgeof the policy process to target initial interviewees, such as officials in specific government departments. These interviewees may be involved in the policy process as individual actors, or they may be members of a collective or corporate actor. Researchers need to classify these individuals by their actions, purposes, resources, and decision-making processes as shown in Table 2. More individuals involved in the policy process can be pinpointed by the snowballing method. The exploration of actor networks does not come to an end until no new actors are mentioned in the interview.

**Table 1:** Steps of PII to identify interest.

	**Step 1**	**Step 2**		**Step 3**	**Step 4**
**Theoretical Underpinning**	Theory of power	Theory of interest		Theory of policy Evaluation	Assumption of intentional action
		Formal	Informal		

**Empirical Evidence**	Document Interview Observation	Document Interview		Document Interview Observation	Step 1+ Step 2+Step 3

**Result**	Actors’ power	Actors’ formal Interest		Policy impact	Formal and informal interests of powerful actor

**Table 2:** Classification of actors from study of Scharpf [9].

	**Aggregate Actors**	**Collective Actors**				**Corporate Actors**
		**Coalition**	**Club**	**Movement**	**Association**	
Actions	Individual	Joint	Joint	Joint	Joint	Organization
Purpose	Individual	Individual	Individual	Collective	Collective	Organization
Resources	Individual	Individual	Collective	Individual	Collective	Organization
Decisions	Individual	Agreement	Voting	Consensus	Voting	Hierarchy

After screening the actors, researchers need to identify who is the powerful one. The potentate can be distinguished by revealing actors’ power. Power “is a social relationship in which actor A alters the behaviour of actor B without recognising B's will [1].” To be noted, actors can either employ power mechanisms directly or utilize resources to carry out threats. In the intense conflict, researchers can observe the power mechanisms they adopt, including coercion, (dis)incentive and unverified information. For such intense interactions, it is possible to find records in the public documents of governments, non-government organizations (NGOs), and companies. In addition to the participants' records, news coverage and academic research of relevant events are invaluable sources of data. Researchers can also adopt semi-structured interviews with key participants and professional observers (e.g., experts) to deeply understand the process of policy implementation. Researchers should focus on information regarding to power mechanisms, such as enacting laws, setting compensation amounts, and promoting expert knowledge. Observations of physical means are also an essential complement to documentary and interview information, and researchers can find traces of the power instruments in their fieldwork, such as fences erected by the government around forests.

When the conflict is not manifest, the powerful actors often use threats to suppress the will of others. For example, states that have a monopoly on violence can issue policies under the guarantee of forceful resources. Experts use their superior position in knowledge to gain the trust of other actors. In such cases, researchers need to observe the coercive, motivational/punitive and informative resources that support the threat mechanism, such as law enforcement agencies, budgets, regulations on rewards and punishments, and expert groups. For information acquisition of power resources, researchers can similarly employ methods such as documents, semi-structured interviews, and observations. However, given the difficulty of data acquisition, triangulation is a data processing method to enhance reliability and validity. The researcher needs to obtain documents from as numerous sources as possible, interview different actors, and validate these data through a comparative approach [17].

#### Step 2: Observing actors’ formal interest

This approach strives to reveal the informal interests of the powerful but does not ignore the significance of formal objectives. On the contrary, this article confirms that actors’ declared missions remain an important driver of forest governance. In the second step, a theoretical definition of interests provides a pathway to explore and categories formal goals. Researchers can find descriptions of organizations’ missions in public documents such as political party manifestos, government reports and NGOs’ statements. Besides this, researchers can interview the members of the organization and inquire about their description of their mission. Interviewees often have a relatively strong desire to express formal goals because these messages align with external expectations of them. Therefore, the key to interviewing is to find subjects who can express their goals clearly and comprehensively, such as the leaders of the organization. After acquthe information, researchers can classify the missions they mention according to ecological and social objectives. Typical objectives relate to biodiversity, forest conservation, carbon reduction, climate change mitigation, transparency, distributional fairness, economic development, etc.

#### Step 3: Revealing formal and informal policy impact

When conflict occurs, actors may express self-interest. Otherwise, it is difficult to observe informal interests directly. Therefore, researchers should seek observable facts on policy impacts in the third step. It is not only to judge whether the designed objectives are achieved [11], but also to observe the actions of targeted groups in response to the intervention [18]. For formal policy impacts, the PII methodology draws on Gibson's sustainability assessment [19] and categorizes eight principles along three dimensions: social, environmental and cross-cutting (justice and adaptability) [20]. As shown in Table 3, these eight principles include socio-ecological systems integrity; resource maintenance and efficiency; meaningful livelihood sufficiency and opportunity; socio-ecological civility and democratic governance; intergenerational justice; intragenerational justice; precautionary practices and adaptability; as well as an integrated approach, simultaneously applying all principles at once, aiming at mutual benefits and multiple gains [19].

**Table 3:** Principles in formal policy impacts Adapted from study of Katja Brundiers and Hallie C. Eakin [20].

**Environmental Principles**	**Justice** • Intra-and inter-generational justice	**Adaptability** • Precaution and adaptability
• socio-ecological systems integrity		
• resource maintenance and efficiency		
**Social Principles**		
• meaningful livelihood sufficiency and opportunity		
• socio-ecological civility and democratic governance		

For informal policy impacts, the theoretical definition of interests emphasizes changes in the powerful actor's control, economic gains and the spread of ideas. As Table 4 shows, researchers should not only focus on the gains of powerful actors in the policy process, but also observe whether actors leverage the policy to gain benefits from external actors. Researchers need to pay attention to documents involving political gains (e.g., division of departmental functions, promotion of officials) economic benefits (e.g., government budgets and NGO fundraising) and strategic profits (e.g., village rules and regulations). For the discovery of specific documents, the researcher needs to combine the theoretical definition of interests and the scenario of the case to find information according to the local context. During the interview sessions, the researcher should conduct semi-structured interviews with multiple actors. Particular attention needs to be paid to other participants' evaluations of the benefits to the powerful. Informal policy impacts may leave physical trails, such as banners set up by the government to promote its own ideology. The researcher also needs to triangulate the information obtained from observations, documents, and interviews.

**Table 4:** Types of informal policy impacts.

**Types of informal policy impacts**	**Observable facts**	**Examples**
Control	The increase in one's control in policy formulation and implementation.	Different ministries compete for turf.
	The extension of one's influence over external actors by using policies.	Bureaucrats gain promotion through nature conservation.
Economic benefit	The increase in one's economic gains in policy formulation and implementation.	Bureaucratic organizations compete for limited budgets in policy making.
	The increase in one's economic benefits from external actors by using policies.	NGOs use nature conservation projects for public fundraising.
Dissemination of ideas	Spreading or defending one's ideas in policy formulation and implementation.	The World Bank spreads neoliberalism in aid policy implementation.
	Disseminating one's ideas to external actors by using policies.	China promotes the message of peaceful rise through the Belt and Road project.

#### Step 4: Deriving powerful actors’ formal and informal interests

In the fourth step, researchers concentrate on the inference of informal interests. Researchers rely on the results obtained from the preceding three steps. Actors may express their informal interests if a conflict occurs. Researchers can record their interests directly and code them along three dimensions: political, economic, and strategic. However, actors often achieve their informal interests without conflict happening. Even in intensive interaction, actors sometimes only claim formal goals and camouflage self-interests. Therefore, it is crucial to infer informal interests by observing policy impacts. The theoretical definition of interest helps us to simplify this process. By observing policy impacts, researchers reveal changes in actors’ control, financial benefits and dissemination of ideas. If there is a significant informal policy impact, researchers can categorize those changes separately as political, economic and strategic interests of the targeted actors.

Finally, through the above steps, researchers can reveal the formal and expressed informal interests of the powerful. Additionally, researchers need iterations of observation when unexpected situations occur. For example, actors perceived as the powerful do not achieve their formal goals in the policy impact. Or actors identified as the powerless gain unexpected formal and informal benefits. Besides the inherent complexity of natural systems, another reason is the ignoration of the power of others. Hence, it requires us to adjust our focus on the actors and to re-follow the four steps of observation. Through the complementary process, researchers can get a credible description of the interests of the powerful.

### A demonstration of the PII method: interests in mortgage financing of forest rights policy

With the reform of collective forest rights, Chinese peasants have gained the right to operate their own forest. With the expansion of the scale of operation, their willingness to take out bank loans has become more robust. However, due to the high risk, financial institutions are less interested in granting loans. To alleviate this contradiction, the local government in Fujian Province relied on state-owned forest farms to intervene in risk prevention and control in the financial sector and committed to the underwriting of mortgaged forest rights, thus facilitating the development of the forest rights mortgage business.

In this case, the research team first interviewed the director of the local forestry bureau and, through the snowball method, interviewed private forest owners, bank directors and managers of state-owned forest farms. By analyzing the decision-making styles of the actors, the research team concluded that the local party committee, the forestry bureau, and the state-owned forestry farm constituted the corporate actors, which is summarized as the local government.

The research team mainly used semi-structured interviews and government document analysis data acquisition methods. It was found that the local government played the role of the powerful in the policy process. Coercive, incentive and informative resources were utilized to obtain the support of banks and foresters. In terms of coercion, the state-owned forest farms committed to using their professional skills to guard the forests under the mortgage. In terms of incentives, the local government contributed RMB 5 million as a guarantee. In terms of information, the state-owned forestry plantations use professional teams to assess the value of the mortgaged forests.

As a powerful actor, the local government's stated mission is to increase financial support for forestry development. This information was supported in interviews and documents. Through interviews with banks and foresters, the research team confirmed the formal impact of the policy. In terms of informal policy effects, other interviewees mentioned that corresponding local government officials were promoted for developing policy innovations, information that is further corroborated by official documents.

Combining the above data, the research team further inferred that the local government is a powerful actor in the forest rights mortgage policy, and its formal goal is to develop financial instruments in forestry. The informal interest of the local governments is the promotion of officials. Under the political mission of developing rural forestry, officials hope to gain political benefits through policy innovation.

## Conclusion

Although the interests are invisible, this study proposes an approach to infer the actors' interests. Based on the theoretical definition of interest, researchers can observe actors’ formal interests based on document and interview, and infer informal interests of actors through the observation of policy impacts. With Power-based Identification of Interest, researchers can reveal the formal and informal interests of powerful actors. The PII approach does not address the powerless actors that have received considerable attention. But it is still significant for forest governance research to expose the interests of powerful actors. Researchers can further infer the influence on powerless actors.

There is scope for further improvement of this approach. The PII provides a theoretical definition of interest. But the classification of formal and informal interests can be supplemented in further empirical studies. Moreover, these abstract concepts need to be further concretized in the institutional setting of the specific case.

## CRediT authorship contribution statement

**Jiacheng Zhao:** Conceptualization, Methodology, Investigation, Writing – original draft, Visualization. **Max Krott:** Writing – review & editing, Supervision. **Jinlong Liu:** Funding acquisition, Supervision. **Lukas Giessen:** Supervision. **Jiayun Dong:** Investigation, Data curation.

## Declaration of interests

The authors declare that they have no known competing financial interests or personal relationships that could have appeared to influence the work reported in this paper.

## Data Availability

No data was used for the research described in the article.
